# Supplementary material for: Mining Transcriptomic Data to Uncover the Association between CBX Family Members and Cancer Stemness
Source: Int J Mol Sci. 2022 Oct 28;23(21):13083. doi: 10.3390/ijms232113083 (PMC9656300; doi:10.3390/ijms232113083)
Supplement: Supplementary file 1 [file ijms-23-13083-s001.zip › Supplementary_Material.pdf]

Supplementary Material

Mining transcriptomic data to uncover the association between CBX family members and cancer stemness

Patrycja Czerwinska, Andrzej Adam Mackiewicz

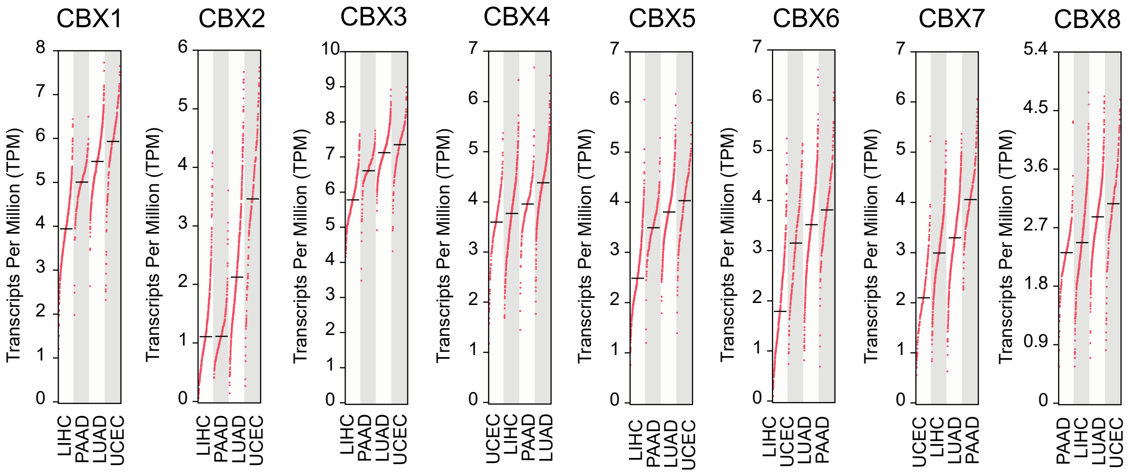

Figure S1. The expression of CBX family members across distinct types of solid tumors using TCGA data in GEPIA2 (http://gepia2.cancer-pku.cn/#general) database.

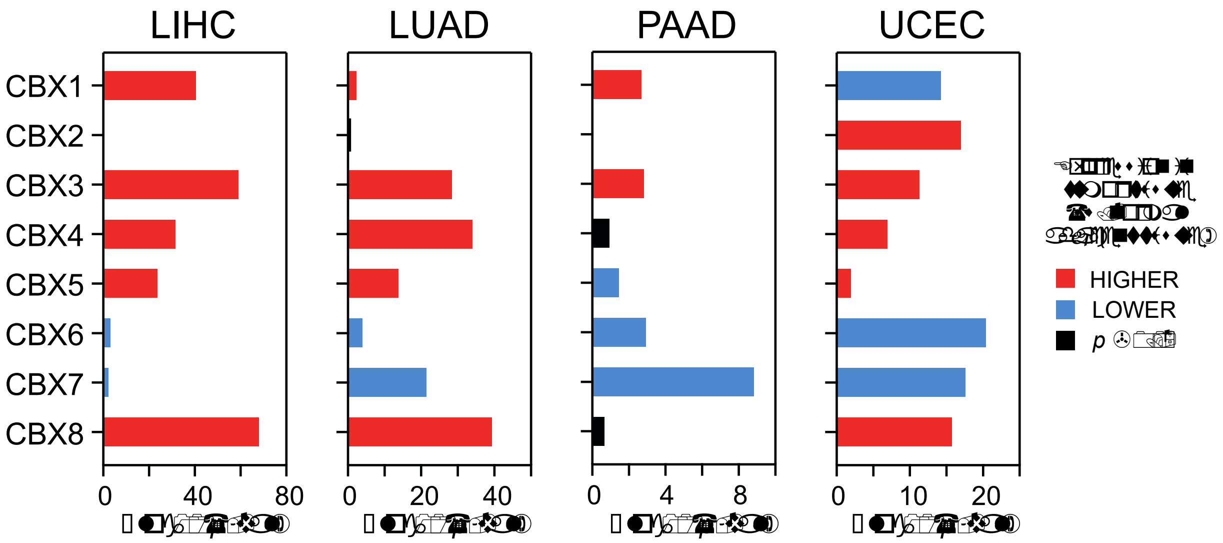

Figure S2. The total protein level of CBX family members in tumor tissues and normal adjacent tissues based on CPTAC data for LIHC, LUAD, PAAD, and UCEC tumors. Red and blue denotes higher or lower expression in tumor tissue, respectively. Statistical significances (−log10(p-value)) for tumor vs. normal tissue comparisons are plotted.

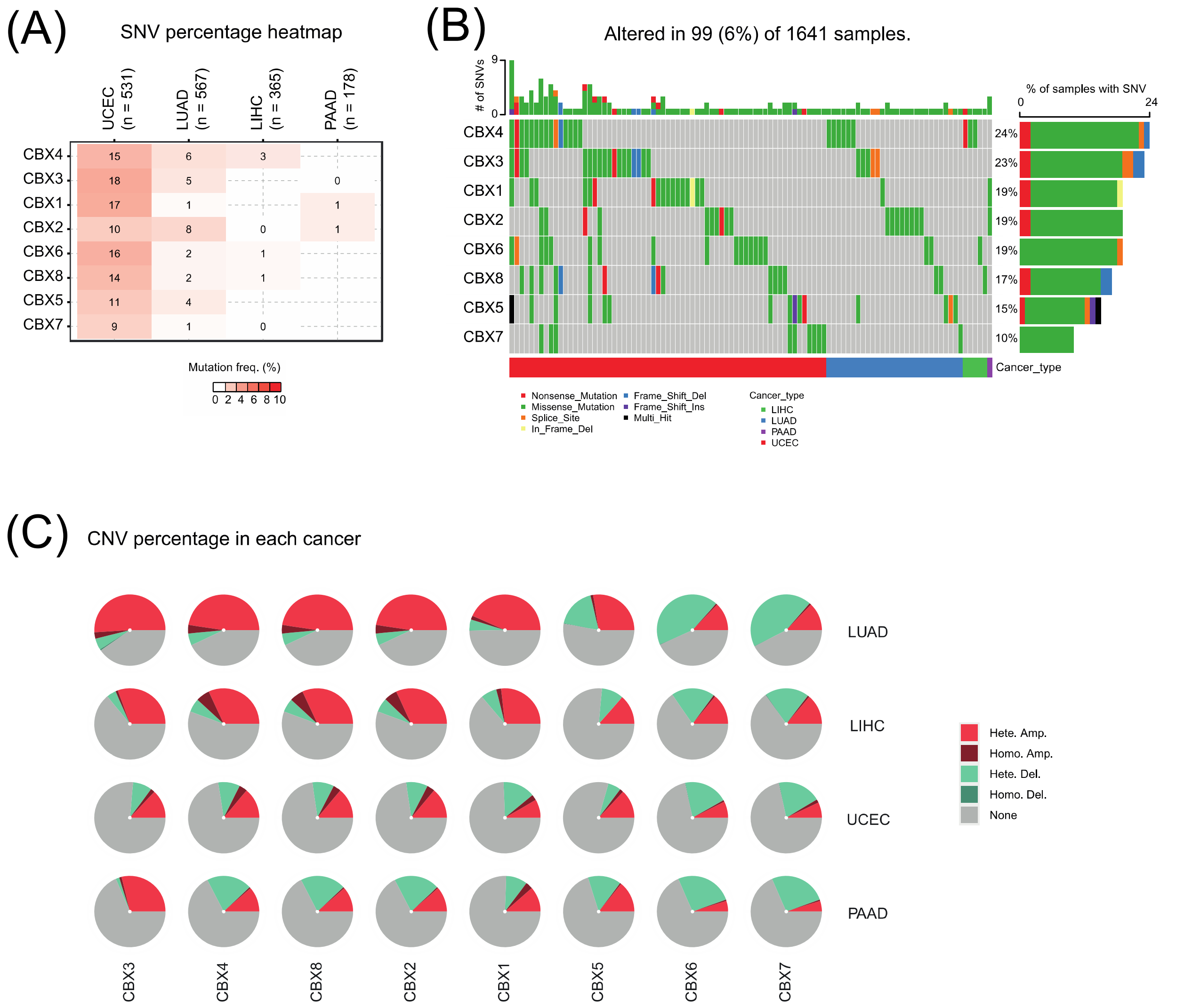

Figure S3. The genomic alterations in CBX family members in TCGA data. (A) The percentage of single nucleotide variations (SNV) in tested tumor types. Numbers on the heatmap denote the number of individuals harboring SNV within distinct CBX members. (B) Types of alterations in CBX family members. Only sampels harboring alterations are presented (99 out of 1641 samples in four tested tumor types). (C) The percentage of copy numer variations (CNV) in CBX family members in TCGA data.

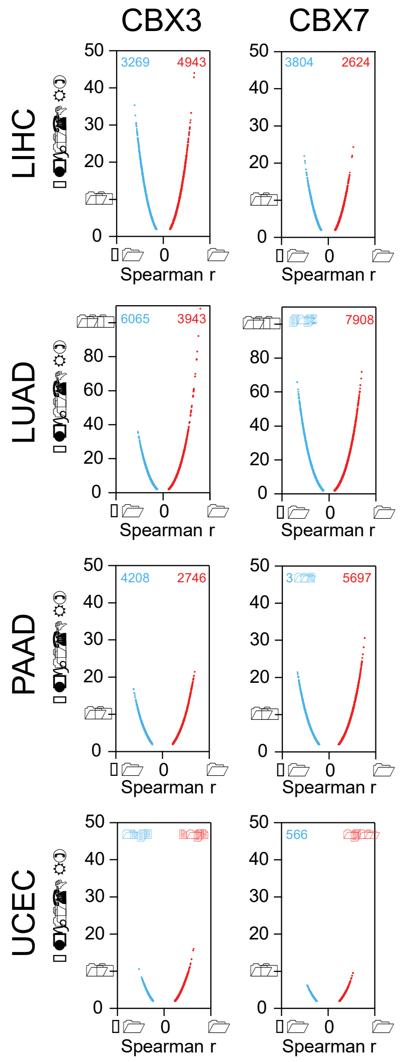

Figure S4. Volcano plots of genes correlated with the expression of CBX3 (left panel) or CBX7 (right panel) in LIHC, LUAD, PAAD, and UCEC tumors. Statistical significance for each correlation (−log10(FDR)) was plotted against the Spearman's correlation coefficient. Blue – genes negatively correlated, FDR < 1%; red – genes positively correlated, FDR < 1%.

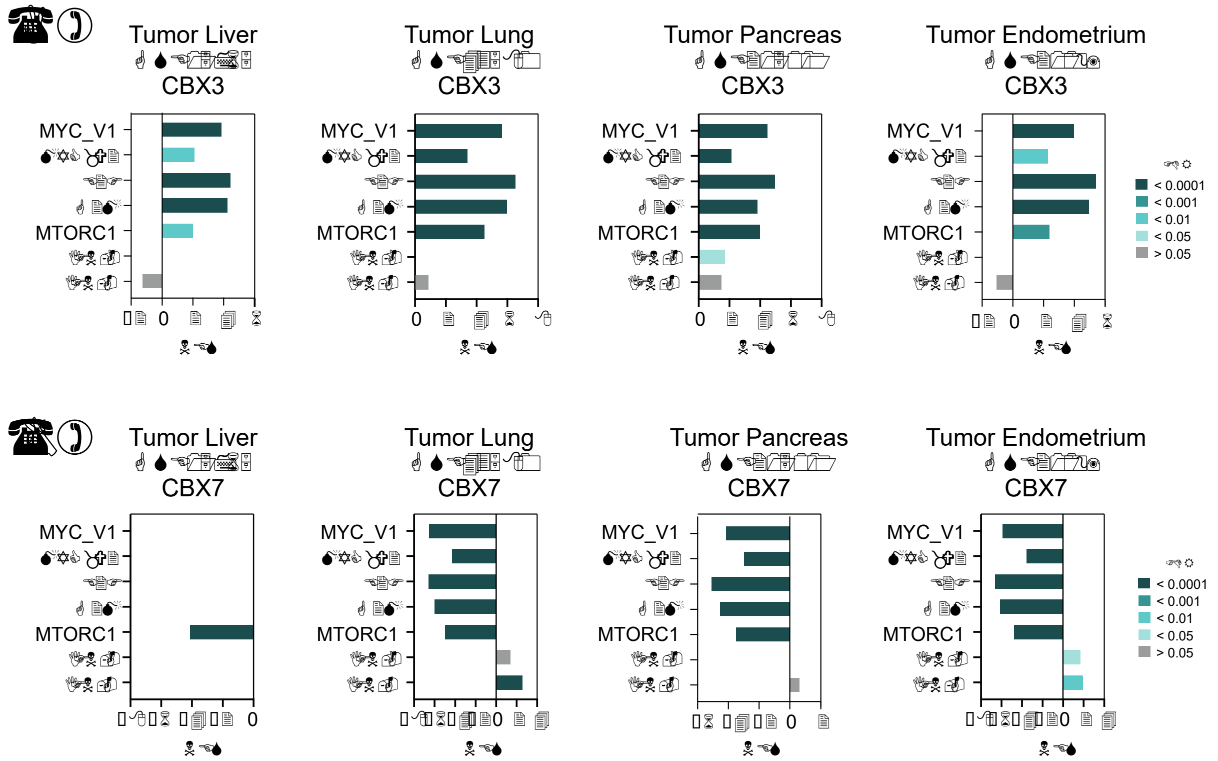

Figure S5. The GSEA of transcriptome profiles associated with the expression of CBX3 or CBX7 in additional GEO datasets. (A) The GSEA of all significantly correlated genes to CBX3 confirmed significant enrichment of c-Myc and E2F transcription factor targets in liver GSE15765, lung GSE43580, pancreatic GSE21501, and endometrial carcinoma GSE2109. (B) On the other hand, the CBX7-associated transcriptome profiles in liver GSE15765, lung GSE43580, pancreatic GSE21501, and endometrial carcinoma GSE2109 are significantly depleted with the „Hallmark of cancer" terms specific for cancer stem cell-like tumors.

Table S1. Datasets from the Oncomine database.

Table S2. Gene lists used to define stemness scores.

Table S3. Additional GEO datasets used in this study.
